# The Unseen Impacts of Human Footprints: How Land Use Reshapes Actinobacterial Communities in the Brazilian Cerrado

**DOI:** 10.3390/biology14040390

**Published:** 2025-04-09

**Authors:** Fernando Gouveia Cavalcante, Leonardo Lima Bandeira, Christiana Mara de Assis Faria, Ariel de Figueiredo Nogueira Mesquita, João Moreira de Matos Neto, Claudia Miranda Martins, Suzana Claudia Silveira Martins

**Affiliations:** 1 National Institute of Science and Technology/Ministry of Agriculture, Livestock and Food Supply (INCT/MAPA), Aracaju 49065-310, Brazil; fernandogouveia.c@gmail.com; 2 Graduate Course of Ecology and Natural Resources, Department of Biology, Federal University of Ceará, Fortaleza 60355-636, Brazil; leonardolbandeira@gmail.com (L.L.B.); fariacma@gmail.com (C.M.d.A.F.); claudiamartins@ufc.br (C.M.M.); suzanac@ufc.br (S.C.S.M.); 3 Graduate Course of Biochemistry, Department of Biochemistry and Molecular Biology, Federal University of Ceará, Fortaleza 60355-636, Brazil; neto.j2802@gmail.com

**Keywords:** biodiversity loss, land use, microbial diversity, preservation units, actinomycetes diversity

## Abstract

Evaluating microbial community changes in soils allows the understanding of ecoevolutionary dynamics, providing information on microbiome responses to anthropological interferences, reduced biodiversity, and climatic changes. The actinobacteria phylum plays crucial roles from an ecological point of view and is the focus of the present study, which tracked changes in the actinobacterial communities from Brazilian Cerrado soil in an environmental protection unit, based on different land uses. The evaluation of 16S rRNA and further taxonomical clustering of operational taxonomic units (OTUs) indicated Actinomycetales as the main order within all land uses, ranging from 45.4–70.1%, with Micrococales and Rubrobacterales being found only in agricultural soils. The evaluation of taxonomical classes revealed Actinobacteria as the main representative in all land uses (45.5–70.4%), with a paired coprevalence of Thermoleophilia (43.4%) in secondary soils, a taxon associated with phosphorus-deficient grounds. The unraveling of families and genera was most challenging due to the OTUs’ dispersion given the volume of data, coupled with a high percentage of unidentified genera (71.6–86.1%), mainly in conserved and preserved areas. The reduced biodiversity, reduced undescribed taxa, and enhanced potentially pathogenic strains in anthropogenic affected sites, which suffer more from climatic changes, denote the harm that human activity causes to microbiological diversity.

## Simple Summary

This study investigated how different land uses in the Brazilian Cerrado, a highly biodiverse region, affect soil microbial communities, particularly actinobacteria, which play vital roles in nutrient cycling and ecosystem health. We aim to understand how human activities, such as agriculture, impact soil biodiversity and microbial diversity. We found that agricultural areas had more potentially harmful bacteria and less actinobacterial diversity compared with preserved areas, which had more unknown and rare species. This study highlights the importance of protecting natural areas to maintain soil health and biodiversity. Climate change, exacerbated by human interference, intensifies biodiversity loss and soil degradation; thus, here, we show the importance of protecting natural areas to mitigate climate change effects and maintain healthy ecosystems, ultimately benefiting society through more resilient and sustainable soils.

## 1. Introduction

Biodiversity is facing an unprecedented crisis, with current extinction rates far exceeding previously reported estimates. The scientific community recognizes five main factors that negatively impact biodiversity: habitat loss, overexploitation of natural resources, biological invasions, pollution, and global climate change [1,2].

Climate change refers to all long-term changes in typical weather patterns, such as temperature, rainfall, and winds, which can intensify seasonal variations leading to extreme events that threaten life and cause great loss of biodiversity [3].

Land use changes have been considered the greatest threat to nature, reducing species abundance and diversity, altering the functionality of ecosystems, and being one of the main causes of global climate change, which is the result of both natural and anthropogenic factors [4].

Soil biodiversity is a key driver of multiple ecosystem functions, playing a crucial role in nutrient cycling, organic matter decomposition, carbon storage, and providing essential support for terrestrial life. All these processes are strongly influenced by the diversity and composition of soil microbial communities, which regulate a wide range of functions, including nutrient availability, plant productivity, and the degradation of toxic waste [5,6].

The profile and composition of the microbiota depend on the physical–chemical characteristics of the soil, such as pH, texture, and organic matter levels, and on environmental factors, such as precipitation, vegetation types, and temperature. Consequently, human interference in the ecosystem, especially changes in land use, has direct impacts on these factors [7,8,9].

Estimating how land use changes affect biodiversity depends on factors such as the study site, the methodology applied, and the taxa under investigation, with these choices leading to different conclusions [1]. For example, on a local scale, land use change is the factor that most impacts the structure of microbial communities, while at larger spatial scales, environmental and soil factors appear to be more influential [10].

Changes in land use create a gradient of human interference in the biological and chemical attributes of the soil. Natural forests are minimally affected compared with grazing areas, which, in turn, are less impacted than areas of intensive cultivation. These impacts are reflected in distinct microbial communities across different land uses [11].

The phyla Proteobacteria, Actinobacteria, Bacteriodetes, and Acidobacteria are dominant in most soil microbial communities, but the composition of the communities varies according to the characteristics of the region, which depend on the type, duration, and intensity of land use [12,13]. Among the phyla mentioned above, actinobacteria represent one of the largest taxonomic units in the bacteria domain, and members of this phylum can be classified into 425 genera, 6 classes, 46 orders, and 79 families [14].

Actinobacteria are naturally occurring and often among the dominant populations of their ecosystems. These are well-adapted to survival in hostile environments, primally due to their ability to form spores and produce a wide diversity of secondary metabolites. However, the composition and abundance of this phylum are regulated by edaphic and climate factors, and its diversity is also influenced by land use changes [15,16,17].

Actinobacteria exhibit diverse morphologies, including coccoid, bacillary, coccobacillary, branched, or fragmented hyphae with spores, may form complex mycelia, and serve various roles as bioremediators, probiotics, producers of hydrolytic enzymes, and clinically important metabolites, such as antifungals, antivirals, antibiotics, and more, though some species are pathogenic to humans, animals, and plants [18,19]. Due to these different morphologies, their life cycles vary, with Streptomyces being the most studied: it begins when a dormant spore germinates in nutrient-rich conditions, forming vegetative mycelium that anchors the bacteria to the substrate, followed by aerial mycelium development when nutrients become scarce. Then, aerial mycelium eventually sporulates, releasing spores to restart the cycle [20]. More recently, it has been discovered that, in specific environmental situations, Streptomyces can abandon their classical lifestyle and adopt an exploratory growth mode through the rapid horizontal growth of vegetative-like mycelium on a solid surface, and is accompanied by the emission of trimethylamine [21].

The Brazilian Cerrado extends over more than 2 million km^2^, making it the second-largest biome in the country, surpassed only by the Amazon. It is recognized as one of the most biodiverse regions in the world. Its vegetation forms a mosaic ranging from savannas to forests, and its soil is mostly acidic and nutrient-poor [7]. This biome has undergone severe deforestation due to agricultural practices and urbanization, leading to extensive degradation.

In Brazil, integrally protected areas aim to preserve nature and are highly restrictive regarding the use of their resources. One type of integrally protected area is National Parks [22]. The Sete Cidades National Park (PNSC) is located in a transition area between the Cerrado and Caatinga in the north of the state of Piauí, Northeast Brazil, and is therefore subject to great environmental heterogeneity, which allows it to shelter a wide diversity of organisms [23]. However, despite Brazilian laws prohibiting its exploitation, the advance of urban and agricultural frontiers threatens its biodiversity [24].

Understanding how changes in land use affect the diversity and structure of microbial communities within and near conservation units is needed, as this knowledge becomes necessary for the development of sustainable land management strategies that aim to reduce soil degradation and loss of biodiversity in this biome. In this context, the objective of this study is to investigate how changes in land use impact the richness, composition, abundance, dominance, and diversity of actinobacterial assemblages in the soils of the PNSC and its surrounding region, and to estimate the loss of diversity in areas with greater human interference. Our hypothesis is that the intensification of land use results in its degradation, causing a loss of biodiversity and negatively affecting the richness and abundance of taxa in soils under greater anthropic pressure. It is also expected that the area under federal protection will harbor a larger contingent of rare taxa.

## 2. Materials and Methods

### 2.1. Study Area

**Figure 1:**
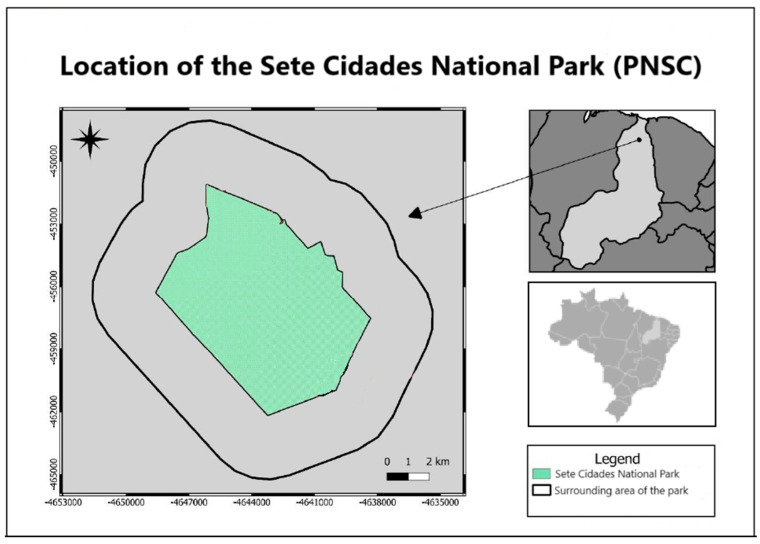
Location of the Sete Cidades National Park (PNSC) and its surroundings.

Sete Cidades National Park (PNSC), located in northeastern Brazil (Piauí State), spans the municipalities of Piripiri, Piracuruca, and Brasileira (04°02′08″ S, 41°40′45″ W) (Figure 1). Established as a protected area under Federal Decree No. 50,744 on 8 June 1961, the park covers 6221 hectares, with a perimeter of 36 km [25]. The region features a sub-humid climate (classified as C2w2A 4a’ under the Köppen–Geiger system), characterized by distinct wet and dry seasons and an average annual temperature of 26.1 °C. Climate data [26] indicate a mean annual precipitation ranging between 1358 mm and 1443 mm, reflecting a slight decrease compared with historical records. Peak rainfall typically occurs between February and April; however, climate change assessments suggest increasing variability in precipitation patterns, including extended dry periods and more intense wet-season events. These changes may have a significant impact on soil moisture retention, water availability, and the overall stability of the ecosystem within the park.

PNSC’s vegetation is highly diverse, comprising six main phytophysiognomies: mesophytic cerrado, flooded gallery forest, dry semi-deciduous forest, typical cerrado, cerrado rupestre, and campo limpo [27]. However, ongoing climatic changes are expected to influence species composition and distribution, potentially affecting ecosystem resilience and biodiversity conservation strategies. These trends underscore the need for adaptive conservation measures to mitigate potential ecological disruptions and ensure the long-term preservation of PNSC’s unique ecosystems [6].

### 2.2. Soil Sampling

Soil sampling was carried out at the end of the rainy season, between 15 May and 1 June 2018, to ensure optimal soil moisture conditions for microbial and physicochemical analyses. Before fieldwork, high-resolution satellite imagery (Rapid Eye) from the Geocatalogue of the Brazilian Ministry of the Environment [28] was used to delineate and map sampling plots within the study area. Preserved areas, defined as regions within the conservation unit without human intervention and characterized by native vegetation in advanced stages of ecological succession, were identified using ArcMap 10 software.

To capture the heterogeneity of vegetation types and land uses, a stratified random sampling method was used. A total of 28 soil samples were collected from 12 distinct landscapes, representing four main land use categories: preserved areas within the conservation unit, characterized by native vegetation in advanced stages of ecological succession; secondary areas with sparse vegetation in intermediate stages of succession; agricultural areas with crops such as corn, beans, squash, and cassava; and conserved areas, consisting of patches of native vegetation in advanced stages of succession, but located outside the conservation unit. Each sample was named according to its land use and the corresponding pixel number, ensuring a systematic and reproducible sampling approach.

At each sampling point, bulk soil samples were collected at a depth of 0–20 cm, following the methodology described by Lucena and collaborators [29]. Each sample consisted of five subsamples collected in a W-shaped transect (20 × 40 m) to account for spatial variability and habitat heterogeneity. A 1 m^2^ quadrant was placed at each vertex of the transect, and the soil was randomly collected within the delimitated area. The five subsamples from each point were aseptically homogenized to form a composite sample, packed in labeled plastic bags, and stored in polystyrene boxes for transport.

The samples were sent to the Laboratory of Environmental Microbiology (LAMAB) of the Federal University of Ceará (UFC) for further analysis. A portion of each sample was stored at −20 °C for DNA extraction and molecular analysis, while the remainder was analyzed for physicochemical properties at the Soil Science Department of UFC.

This sampling strategy was designed to address the potential impacts of vegetation heterogeneity and land use on soil microbial communities, particularly actinobacteria, which play a critical role in nutrient cycling and ecosystem resilience. Given the increasing variability in precipitation patterns and extended dry periods associated with climate change, understanding these dynamics is essential for developing adaptive conservation strategies in the face of ongoing environmental changes.

### 2.3. Physicochemical Analysis of Soil, and Amplification and Sequencing of 16S rRNA Gene

To investigate the relationship between soil properties and the composition of the microbial community, physicochemical analyses were performed following Teixeira and collaborators [30]. The key parameters included electrical conductivity, pH, macronutrients (calcium, magnesium, sodium, potassium, and available phosphorus), micronutrients (iron, copper, zinc, and manganese), organic carbon, nitrogen, and soil texture (% clay, % silt, and % sand). Three replicates were tested for each soil sample analysis. The sample results were grouped by land use and the means of each parameter were compared using one-way ANOVA after performing tests of normality and homoscedasticity.

All molecular biology and bioinformatics analyses were conducted according to Bandeira and collaborators [31]. Total DNA was extracted from 0.25 mg of each soil sample using the DNeasy PowerSoil Kit (Qiagen, Germantown, MD, USA). The DNA concentration and quality were assessed using the Qubit dsDNA BR Assay Kit (Thermo Fisher Scientific, Waltham, MA, USA). The V3–V4 region of the 16S rRNA gene was amplified using primers 314F (5′-CCTACGGGNGGCWGCAG-3′) and 805R (5′-GACTACHVGGGTATCTAATCC-3′), generating amplicons of approximately 491 bp [32]. The PCR program consisted of an initial denaturation step at 95 °C for 3 min, followed by 27 cycles of denaturation at 95 °C for 30 s, annealing at 55 °C for 30 s, and extension at 72 °C for 30 s, with a final extension at 72 °C for 5 min. Amplification reactions were performed on a Veriti^TM^ Thermal Cycler (Applied Biosystems Inc., Foster City, CA, USA).

Amplicons were purified using Agencourt AMPure XP magnetic beads (Beckman Coulter Life Sciences, Indianapolis, IN, USA) to remove primer dimers and other small fragments. Indexing was performed using the Nextera XT Index Kit v2 (Illumina, San Diego, CA, USA), following the manufacturer’s protocol. Indexed libraries were purified again with Agencourt AMPure XP beads to ensure the removal of residual primers and very small fragments. Library quantification was performed using a KAPA Library Quantification Kit-Illumina/Universal (Roche, Indianapolis, IN, USA) on a QuantStudio 3 Real-Time PCR system (Applied Biosystems).

An equimolar pool of DNA libraries was prepared by normalizing all samples to 4 nM. Sequencing was performed on the Illumina MiSeq platform (2 × 250 bp paired-end reads) at BPI—Laboratório de Biotecnologia, Pesquisa e Inovação, Brazil.

### 2.4. Bioinformatics and Data Analysis

Bioinformatics analysis was conducted using QIIME 2 (2019.1 release) [33]. Raw sequence data were demultiplexed and quality-filtered using the q2-demux plugin. Denoising was performed with DADA2 (https://github.com/benjjneb/dada2, accessed on 6 April 2025) [34] to generate amplicon sequence variants (ASVs). The ASVs were aligned using MAFFT (https://mafft.cbrc.jp/alignment/server/index.html, accessed on 6 April 2025) [35] and used to build a phylogenetic tree with FastTree2 (https://github.com/morgannprice/fasttree, accessed on 6 April 2025) [36]. Taxonomic classification was performed using the q2-feature-classifier [33] against the Greengenes 13_8 99% OTUs reference database [37].

The raw data used for this work can be found in the NCBI SRA database in BioProject PRJNA1009150, with the following accession numbers: Agriculture (SRS23561114, SRS23561115, SRS23561126 and SRS23561131 to SRS23561135); Secondary (SRS23561122 to SRS23561125, and SRS23561127 to SRS23561130); Conserved (SRS23561116 to SRS23561121, SRS23561136 and SRS23561137); and Preserved (SRS18980979 to SRS18980982).

To estimate community diversity, the parameters abundance, richness, dominance, Shannon index, and evenness were used, considering the land use categories and the different taxonomic levels studied. To calculate the Shannon and evenness indices, the data were normalized as established by Kim and collaborators [38] because they were samples of different sizes. Multivariate NMDS analysis was used to obtain the pattern of similarity between communities. The adequacy of the model was measured by the Stress parameter, and the adopted value was ≤0.2. To evaluate the influence of soil chemical factors on community structure, a multivariate redundancy analysis (type II scaling) was used [39]. Diversity indices and multivariate analyses were performed using PAST software version 4.17.

## 3. Results

### 3.1. Soil Physicochemical Properties

The PNSC soil sample properties are shown in Table 1, presenting the key physicochemical properties: electrical conductivity, pH, macronutrients (calcium, magnesium, sodium, potassium, and available phosphorus), micronutrients (iron, copper, zinc, and manganese), organic carbon, nitrogen, and soil texture (% clay, % silt, and % sand).

### 3.2. Distribution of Actinobacterial OTUs

**Figure 2:**
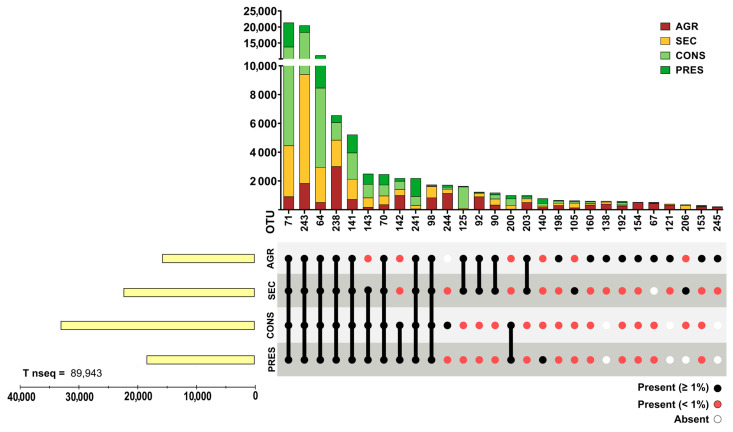
Distribution of highly abundant (with relative abundance ≥ 1% in at least one land use type) actinobacterial OTUs. The upper panel presents bar graphs for data grouped by OTU while the bottom panel highlights the presence (≥1% or <1%) or absence of a given OTU by land use. Left bar graphs present the grouped number of reads of all highly abundant OTU divided by land use.

**Figure 3:**
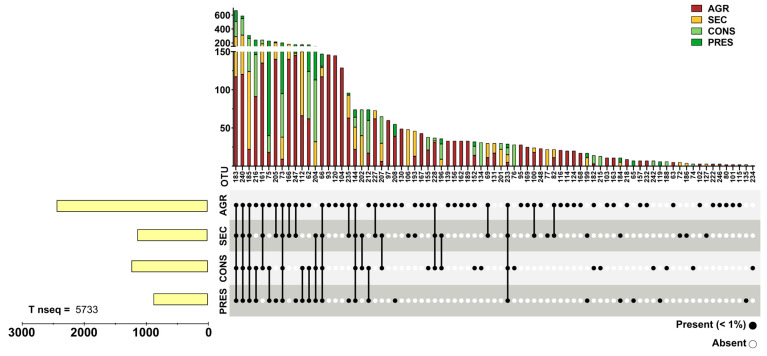
Distribution of sparse (with relative abundance < 1% in all land uses) actinobacterial OTUs. The upper panel presents bar graphs for data grouped by OTU while the bottom panel highlights the presence or absence of a given OTU by land use. The left bar graphs present the grouped number of reads of all sparse OTUs divided by land use.

Prokaryotic 16S rRNA genes were obtained through NGS from 28 soil samples. After actinobacterial data filtering of OTUs, a total of 93,236 sequences were retained. The OTUs were distributed in four different land uses and distributed as follows: 18,289 sequences in agricultural soils (19.61%), 23,577 in secondary soils (20.79%), 34,443 in conserved soils (36.93%), and 19,377 in preserved soils (22.67%). A total of 107 OTUs were observed and unevenly distributed across land uses, where agricultural samples presented 85 OTUs (83.33%), 60 secondary OTUs (57.84%), 55 conserved OTUs (53.92%), and 47 preserved (46.07%), with a certain degree of overlap in each land use. OTUs with a relative abundance within each land use type greater than 1% were cataloged, representing almost 90% of the total acquired sequences (Figure 2), although they accounted for only 25.92% (28) of the total observed OTUs. Conversely, 80 OTUs (73.83% of total) comprised only slightly more than 10% of the total sequences (Figure 3). Detailed information about each OTU is provided in Supplementary Table S1.

### 3.3. Relative Abundance of Taxa

**Figure 4:**
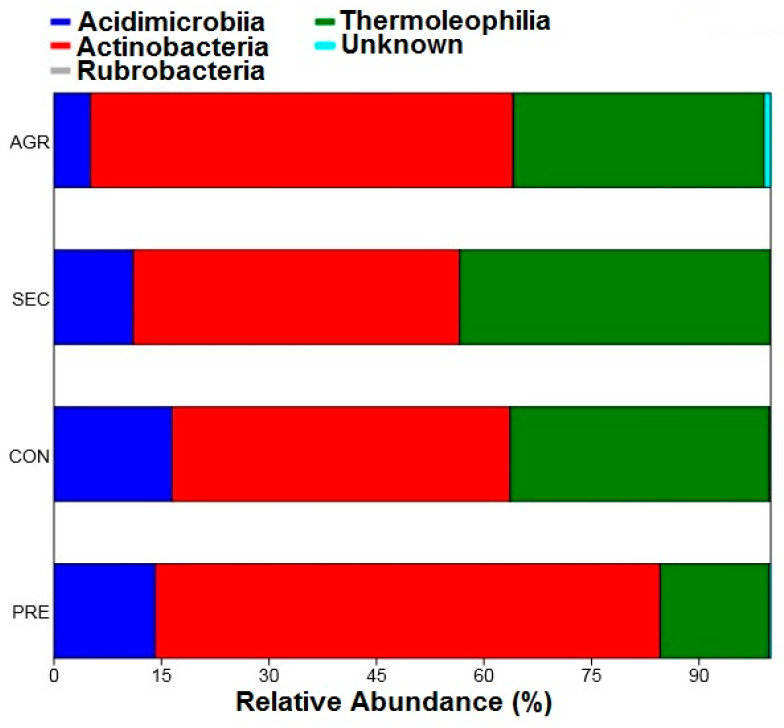
Relative abundance of classes belonging to the Actinobacteria phylum of the PNSC and surrounding region.

Relative abundances were determined for the taxonomic levels of class, order, family, and genus, considering the different land uses. The results show that the Actinobacteria class was the most abundant in all soil categories studied, representing up to 70% of the total in the preserved area (Figure 4). The Thermoleophilia class also stood out as the second most abundant, corresponding to up to 43% of the total for the secondary area. On the other hand, the Rubrobacteria class had the lowest relative abundance (0.03%), being present only in a portion of the agricultural area (A13), making it a rare taxon. The Acidimicrobiia class appeared in the four use categories, but appeared to be more affected by human activity, having a lower relative abundance in the agricultural area (5%) than in the preserved area (16%).

**Figure 5:**
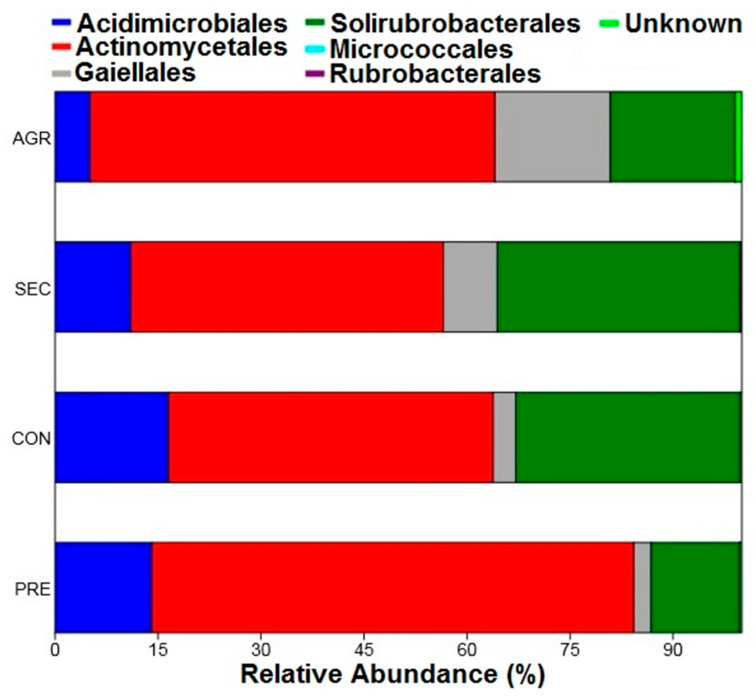
Relative abundance of orders belonging to the Actinobacteria phylum of the PNSC and surrounding region.

Regarding the relative abundances of the orders, the results followed the same pattern presented by the classes, with prevalence of the order Actinomycetales, followed by Solirubrobacterales (Figure 5). For this taxonomic level, the order Micrococcales stands out, which exhibited a relative abundance of 0.016% of the agricultural area and was present only in one sample plot (A34), indicating that it was a rare taxon.

**Figure 6:**
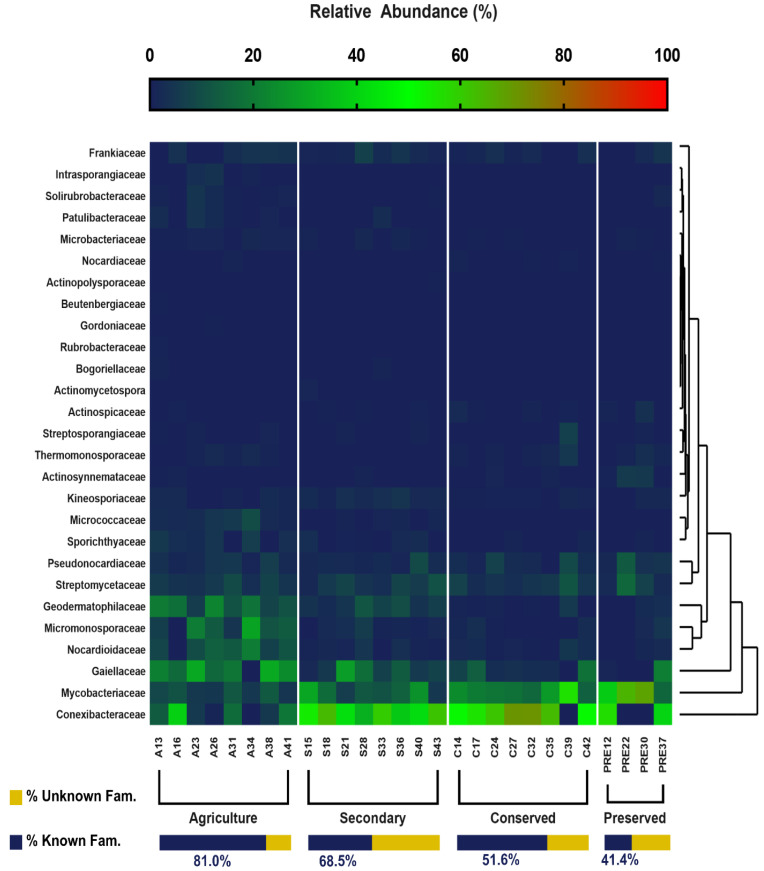
Relative abundance of families belonging to the Actinobacteria phylum of the PNSC and surrounding region.

Further investigation at the family and genus levels revealed more details and key differences between land uses. Regarding the known Actinomycetota families, 27 families were identified (Figure 6), of which 25 were present in the agricultural area, 21 in the secondary area, 18 in the preserved area, and 17 in the conserved area. The most abundant families were Conexibacteraceae, obtaining a maximum value of 32% in the secondary area. The families Mycobacteriacae and Gaiellaceae were also abundant, with maximum values corresponding to 18.34% (preserved area) and 16.59% (agricultural area). Agricultural soil samples revealed a more uneven and random distribution of families. The rarest taxa were those belonging to the families Intrasporangiaceae (0.78%), Actinomycetospora (0.09%), Rubrobacteraceae (0.03%), Actinopolysporaceae (0.02%), Gordoniaceae (0.01%), and Beutenbergiaceae (0.01%). It is also important to highlight the percentage of unidentified taxa, mainly in the soils of the forested areas, which was 58% in the preserved area and 48% in the conserved area (Figure 6), which demonstrates the potential of these areas for the discovery of new species.

**Figure 7:**
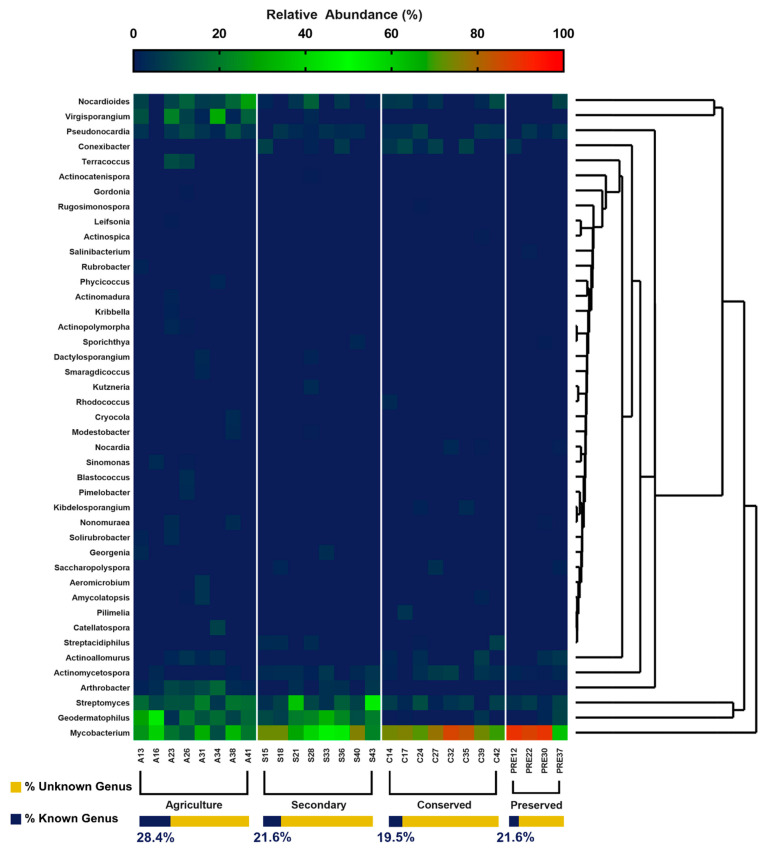
Relative abundance of genera belonging to the phylum Actinobacteria of the PNSC and the surrounding region.

Agricultural soils showed the greatest percentage of known genera, while preserved soils exhibited the lowest. The analysis of genera resulted in 43 distinct genera, of which 30 were present in the agricultural area, 17 in the secondary and conserved areas, and 13 in the area under federal protection (Figure 7). *Gordonia*, *Actinocatenispora*, *Leifsonia*, *Rugosimonospora*, and *Actinospica* genera were the rarest found, corresponding to 0.01% of the genera obtained. The most abundant genera among the areas were *Mycobacterium* (85% in the preserved area), *Geodermatophilus* (17.6% in the agricultural area), *Streptomyces* (13.6% in the agricultural area), and *Nocardioides* (10.8% in the agricultural area). The genera *Actinomadura*, *Actinopolymorpha*, *Aeromicrobium*, *Blastococcus*, *Catellatospora*, *Cryocola*, *Gordonia*, *Kribella*, *Leifsonia*, *Phycicoccus*, *Pimelobacter*, *Rubrobacter*, *Sinomonas*, *Smaragdicoccus*, *Solirubrobacter*, and *Terracoccus* occurred only in the agricultural area.

In native forest areas, the genera *Rugosimonospora*, *Rhodococcus*, *Pilimelia*, *Kibdelosporangium*, and *Actinospica* occurred only in the conserved area, the genus *Salinibacterium* only in the preserved area, and the genera *Kutzneria* and *Actinocatenispora* only in the secondary area. It is worth noting that the calculation for the relative abundances of the genera did not consider the unknown genera, since they corresponded to up to 86% of the total in each use class, which would make most of the other taxa rare. The results for genera reinforce those obtained for families in which the areas under conserved forests shelter a larger contingent of uncatalogued taxa, denoting their potential for the discovery of new species.

### 3.4. Diversity Indices

The diversity indices varied between taxonomic levels and land uses, with higher values in the area within the PNSC. The Shannon index (H) obtained its highest value in the soil of the park, reaching 4.148 for the family taxonomic level. On the other hand, the lowest value was obtained in the conserved area for the class taxonomic level (1.556).

Evenness, which is a descriptor of the stability of a community profile, also obtained higher values in the soil sampled within the UC, obtaining a maximum value of 3.517 considering the bacterial families studied, and the lowest value was found considering the classes in the conserved area (1.58). Agricultural areas also tended to have lower equilibrium values than the others.

**Figure 8:**
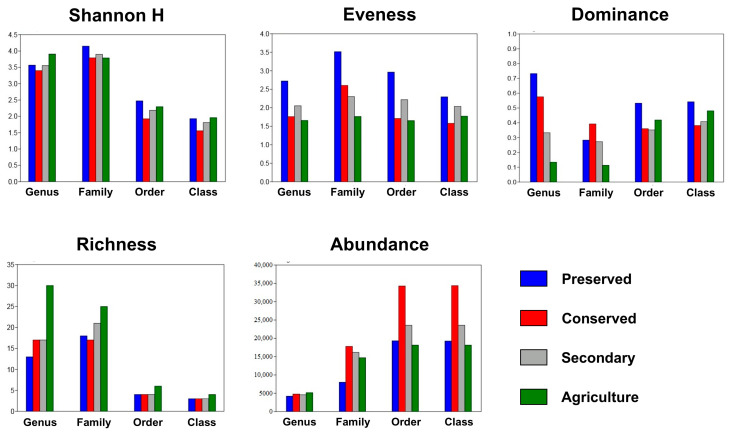
Shannon indices and evenness, dominance, richness, and abundance of bacterial communities across land uses at different taxonomic levels. PRE—preserved; CON—conserved; SEC—secondary; and AGR—agriculture.

The dominance index was also determined for the land use categories, as well as for the four taxonomic levels evaluated. Maximum and minimum dominance values were observed in the genera, with values equal to 0.7325 for the preserved area and 0.1136 for the agricultural area. The family and genus taxonomic levels showed greater discrepancy between the dominance indices considering the types of land use than the class and genus levels (Figure 8).

The richness and abundance parameters showed a peculiar pattern, because as the taxonomic level became more specific, richness increased, and abundance decreased. Greater richness was found in the agricultural area at all taxonomic levels analyzed. On the other hand, greater abundance was associated with the soils of the conserved area.

It can be noted that the abundances of classes and orders obtained for the respective land use categories were practically the same, as shown in Figure 8. However, the abundances for more specific taxonomic levels decreased from 20,000–35,000 sequences at the class and order levels to less than 5000 in the genera.

### 3.5. Multivariate Analysis

#### 3.5.1. Nonmetric Multidimensional Scaling

Nonmetric multidimensional scaling (NMDS) was used to assess the distribution patterns of bacterial abundances, together with soil chemical parameters. The results were divided by taxonomic level.

**Figure 9:**
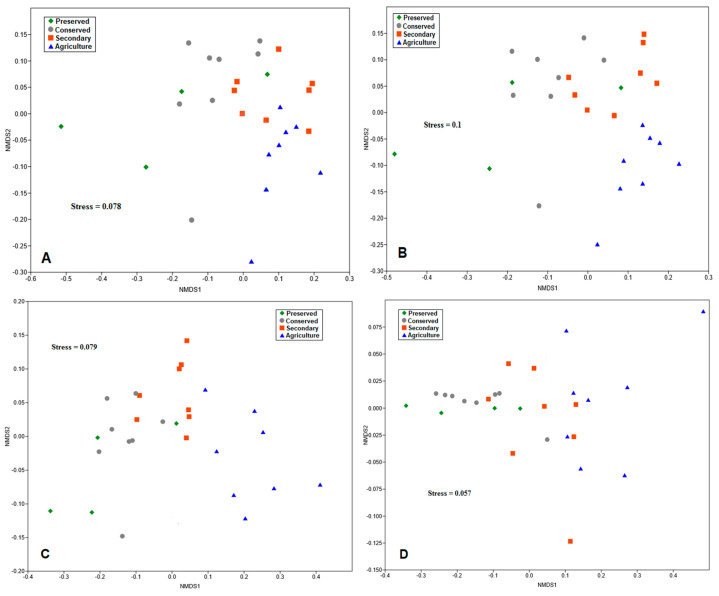
Graphs of the nonmetric multidimensional scaling (NMDS) analysis of the soil bacterial communities of the PNSC and surrounding region at different taxonomic levels: (**A**) class; (**B**) order; (**C**) family; and (**D**) genus.

For all analyses, Stress, which was the measure used to verify the adequacy of the model, was below 0.2, indicating good adequacy. For the class taxonomic level, the points sampled in the forested areas had greater data heterogeneity (Figure 9A). On the other hand, the points in the agricultural area were more grouped, suggesting greater similarity between the communities in this area.

At the order taxonomic level, microbial communities in native forest plots exhibited greater clustering, indicating higher levels of similarity among them (Figure 9B). However, the plots in the preserved area showed greater heterogeneity. For this taxonomic level, the agricultural area was also more isolated from the others, suggesting a distinct pattern compared with the other land uses.

Scaling the data at the family level (Figure 9C) showed a pattern very similar to that obtained for orders. The bacterial communities at the genus level presented a quite different pattern from the previous ones, since the points corresponding to the plots of the areas under preserved forest were grouped closer together (Figure 9D), revealing similarity between the communities in these areas. In contrast, the plots of the secondary and agricultural areas presented greater variability in their data.

#### 3.5.2. Redundancy Analysis

**Figure 10:**
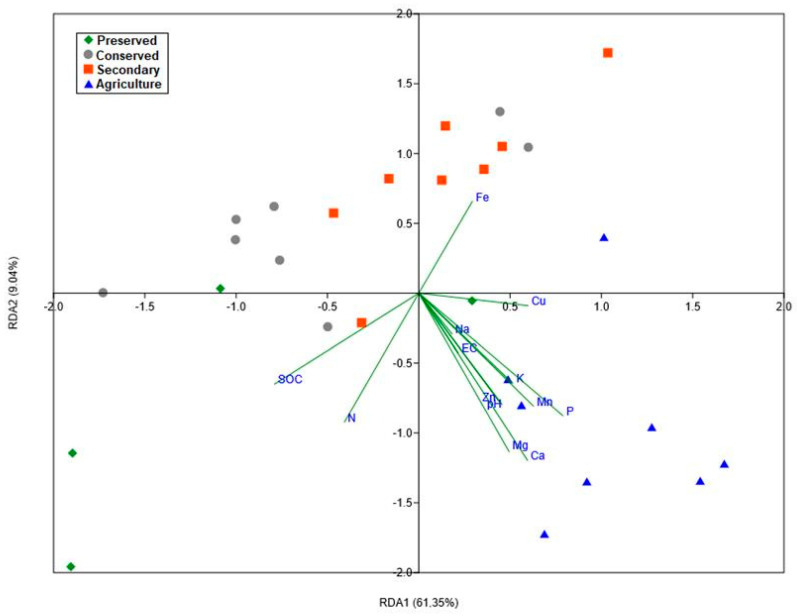
Graph of the redundancy analysis of the abundances of the Actinobacteria phylum and chemical parameters of the soil of the PNSC and the surrounding region. (R^2^ = 0.7314; R^2^adj = 0.482; F = 2.932; *p* = 0.007).

For this analysis, only the data related to bacterial families met the test assumptions. The redundancy analysis confirmed the significant influence of environmental variables on the structure of the bacterial community, and the estimated model was able to explain up to 73% of the variance in abundances. It can be noted that the environmental variables with the greatest influence were soil macronutrients (N, P, Mg, and Ca) and total organic carbon. The sodium content had less influence (Figure 10). The model also indicated that the plots in the agricultural area were more isolated from the others, indicating a more specific community pattern for this land use. The plots sampled within the Park displayed higher variability among themselves, further supporting the findings from the earlier analysis.

## 4. Discussion

This study demonstrated the influence of land conversion on microbial structure and diversity, with a tendency toward loss of diversity in areas under greater anthropogenic pressure. For comparison purposes, higher values in these indices indicated greater community integrity and stability [40]. Therefore, the results of this study reinforce the importance of creating and maintaining protected areas as a strategy to conserve biodiversity in the face of anthropogenic impacts.

The Actinobacteria class is recognized as dominant in most soils, a fact that was also corroborated in this study, where it represented the highest relative abundance among the classes studied. The Thermoleophilia class comprises strains of the Actinobacteria phylum with thermophilic characteristics that are found abundantly in soil and hot springs [41]. The plots in the secondary area had a higher relative abundance (43%) for strains of this class, which may be related to the low vegetation cover, which results in higher soil temperatures and selecting more adapted communities.

The Acidimicrobiia class comprises acidophilic and moderately thermophilic microorganisms [42]. The results show that this class was less abundant in the agricultural area, where soil pH is more alkaline than that under the other use classes. This suggests that strains of this class are better adapted to conditions of greater acidity. The soil of the plots in the conserved area presented the lowest pH values, together with the highest relative abundance of this class.

The Rubrobacteria class had a relative abundance of only 0.03%, which can be classified as rare (<0.1%) and was present only in agricultural areas. This class inhabits terrestrial and marine environments, hot springs, and volcanic soils, reflecting its high diversity and adaptability [43]. This class was also found in soils from arid regions of Australia with relative abundances ranging from 2.6 to 10.2% [44], suggesting a rather random and diverse distribution pattern.

The distribution of taxa at the order level was similar to that presented for the classes. For both levels, only the agricultural area presented rare taxa (Rubrobacterales and Micrococcales). This pattern was also observed at the family and genus taxonomic levels. Figure 6 and Figure 7 clearly show a greater number of taxa in the agricultural area compared with the other uses. This is reinforced in Figure 8, where the taxon richness is clearly higher in the agricultural area plots.

It is expected that the soil in conserved forests will present greater richness and, consequently, a greater contingent of rare organisms [45], which does not reflect the results. However, this pattern is presented by a specific phylum and may not apply to the entire microbial population. Additionally, the Actinobacteria phylum is known for its resistance and resilience to environmental disturbance [15]. It is also worth noting that the richness parameter should be avoided to quantify and compare microbial diversity with metagenomic data, as they are not capable of estimating the absolute number of rare species [46].

From another perspective, molecular analyses at lower taxonomic levels show a huge contingent of unidentified organisms in areas under conserved forests, corresponding to up to 86% of the sequences read for the genus taxonomic level. This percentage not only reflects the limitations of the technique used [47], but also the possibility that the organisms in this biome have not yet been cataloged and therefore remain unknown.

Among the genera found in the preserved area, the following stand out: *Mycobacterium*, which harbors human pathogenic species such as *M. leprae* and *M. tuberculosis* [48]; *Actinoallomurus*, which may present antimicrobial activity and biosynthesis of numerous secondary metabolites [49]; *Pseudonocardia*, which has great ecological importance, for example, in establishing symbiosis with ants, and biotechnological importance, being able to act in agriculture and the bioremediation of areas contaminated by alkylpyridines, organochlorines, and plastics, and in pharmaceutical research due to its antimicrobial and neuroprotective activity [50]; and *Streptomyces*, a genus that needs no introduction due to its prolific production of antibiotics, antitumor compounds, biofilm inhibitors, antiparasitics, bacterial toxin production inhibitors, and antioxidants [51], but also interacts with plants, animals, and other microorganisms in their diverse habitats [52].

In addition to *Mycobacterium*, *Streptomyces*, *Actinoallomurus*, and *Pseudonocardia* being present in the preserved area, the conserved area also harbors *Nocardioides*, which can degrade a variety of recalcitrant pollutants, such as aromatic compounds, hydrocarbons, haloalkanes, nitrogen heterocyclics, and polyesters [53]; *Conexibacter*, which can act as a phytopathogen, is also capable of metabolizing small-molecule organic acids [54] and producing α-pyrones, which function as sulfate shuttles [55]; and *Actinomycetospora*, which is commonly found in soils and in association with plants and animals [56].

It is also important to highlight that the contingent of unidentified taxa, mainly in areas under conserved forest, limits the estimate of the real diversity that is reflected in the indices, as they may represent rare and uncommon taxa that generally represent a reservoir of genetic and functional diversity and may become numerically important under conditions of environmental change [57].

The results show greater dominance in areas under forest in a more advanced successional stage, especially considering bacterial genera. Dominance is a widely studied parameter due to its ecological implications. Greater dominance in a given community is associated with a greater number of rare species, although with a lower relative abundance [58]. These rare organisms are fundamental for the maintenance of soil and ecosystem functions and are of great relevance for biogeochemical processes [59].

As already stated by other studies, crop-growing areas have also shown an increased number of specific taxonomic units, coupled with elevated richness and diversity, which is explained primarily by reduced soil acidity, leading to a more near-neutral pH [60]. A widespread practice among local farmers of the studied areas involves the combustion of natural vegetation prior to cultivation establishment, which allows for basic ions from ashes to seep into the soil, increasing its pH [61]. Conversely, the forest has more acidic soils, a result of multiple factors, such as the presence of nitrification bacteria and the immobilization/harvest of cations promoted by tree roots [62]. Soil acidity also acts as a natural microbiome regulator, shaping the microbial community by preventing the development of intrusive bacteria, while promoting the development of nutrient cycling and mineral weathering bacteria that enhance nourishment sources on soil [63,64].

Agricultural soil samples revealed a more uneven and random distribution of families. The Nocardiodaceae family was more prevalent in agricultural soils, being associated with pathogenesis in humans, encompassing some dangerous strains, such as *Nocardia farcinica*, *Nocardia abscessus*, and *Nocardia brasiliensis* [65]; and plants, causing phytopathologies such as false broomrape [66]. On the other hand, they can establish symbiotic relations with those organisms by producing plant growth promoters [66] or inhibiting phytopathogenic fungi colonization [67].

The agricultural area had 16 additional genera compared with the other use categories, two of which had a frequency lower than 0.01%. It obtained the highest Shannon index for the genera (3.906) and greater taxonomic richness at all levels studied, evidencing greater diversity than the other land use categories. This conclusion would be valid if we considered only the identified sequences, but the contingent of unidentified sequences from the conserved areas cannot be ignored, which changes this perspective, as previously mentioned.

Despite being established in 2004, the DNA barcoding technique still has limitations in terms of the coverage of species cataloged in its databases [68]. This is even more pertinent when dealing with such an abundant and diverse biological group as microorganisms. Our results reflect this, because, as the specificity of the taxa increased, the proportion of identified sequences decreased drastically (Figure 8).

Evenness is a diversity metric related to the stability of a community, so higher values are associated with greater stability [69]. This study showed that, at all taxonomic levels evaluated, evenness was higher within the area under federal protection. This demonstrates the effectiveness of conservation units in maintaining biodiversity, reinforcing the importance of maintaining and expanding these areas.

On the other hand, areas under agricultural cultivation showed lower evenness at the taxonomic levels of order, family, and genus, which demonstrates the negative impact of human activity in land conversion on soil diversity. The conversion of native forests to agricultural fields causes profound changes in microbial composition [70,71], which would consequently affect the distribution of abundances and community stability.

The plots of the conserved area were more exposed to disturbances from urban expansion and agricultural activities, which are common in the studied area, since they are not protected by federal law. This land use class showed lower diversity indices than those in the conservation unit, even though the landscapes are similar. This can be explained by habitat fragmentation [72] outside the CU, as sample plots of this land use class actually comprise forest fragments.

This result further reinforces studies linking habitat fragmentation with biodiversity loss [73,74]. The effects of fragmentation may be more severe if we consider the local area’s abiotic conditions, which are characterized by extended periods of drought and sandy soils with low nutrient availability. It is also important to highlight the greater heterogeneity of the secondary area compared with the conserved area.

The secondary area is characterized by landscapes with more open and spaced vegetation, which are typical of Cerrado in the semiarid region [75] and comprises a larger area than dense forests, and is therefore less affected by the effects of habitat fragmentation. Furthermore, secondary succession is developing, which leads to a greater release of root exudates that stimulate the root-associated microbiota, modulating the dynamics of microbial communities [76].

Multivariate analysis showed high heterogeneity of bacterial communities and soil chemical parameters in the plots within the PNSC, which reflects the physiographic heterogeneity of the region. At the taxonomic levels of class, order, and family, the plots in the agricultural area appear more grouped together, suggesting a pattern distinct from those of the other land use categories, which reflects the direct impact of human activity on the microbial community. Although a distinct pattern was observed in the genera, the contingent of unidentified taxa influenced the analysis.

The redundancy analysis showed a significant influence of soil parameters on the structuring of bacterial families, with macronutrient levels (N, P, Mg, and Ca) being the most important factors. Since the soil in this region has low nutrient availability, as previously mentioned, it is assumed that the limitation of these resources is the most active soil factor in modulating the microbiota. The availability of resources has also been a factor associated with the regulation of plant communities in Cerrado [77], and this is directly related to the microbiota due to the interactions between plants and microorganisms.

## 5. Conclusions

This study examined how land use changes impact actinobacterial communities in the Brazilian Cerrado, a biodiverse yet threatened biome. Actinobacteria dominated all sites (45.5–70.4% abundance), but preserved and conserved areas, such as Sete Cidades National Park (PNSC), exhibited greater evenness and stability in microbial communities (as indicated by higher Shannon diversity indices), while also having exceptional diversity, including up to 86% of unidentified genera. This highlights the Cerrado’s vast unexplored actinobacterial diversity. In contrast, even though they showed higher actinobacterial richness, agricultural soils showed reduced biodiversity, uneven community structures, and an increase in potential pathogens like *Nocardia*, underscoring the destabilizing effects of human activity.

The findings emphasize the urge to protect the Cerrado’s soil microbiome, which serves as both a genetic reservoir and a buffer against ecosystem degradation. Conservation policies must prioritize PNSC and its buffer zones to avoid the loss of biodiversity caused by agricultural and urban expansion.

Finally, we call for further research to explore the functional roles of unidentified microbial taxa and their potential contributions to ecosystem services. Understanding these microbial communities in more depth could inform sustainable land management practices and contribute to global efforts to mitigate biodiversity loss in the face of climate change and anthropogenic pressures. The Cerrado soil microbiome remains a largely untapped resource, and its preservation is essential for the future of this globally significant biome.

## Figures and Tables

**Table 1:** Soil physicochemical properties. Results are shown as average ± standard deviation. Letters represent statistically distinct results.

Parameters	Land Uses			
	PRE	SEC	AGR	CONS
pH	4.76 ± 0.33 ^a^	4.76 ± 0.39 ^a^	5.37 ± 0.30 ^b^	4.72 ± 0.21 ^a^
EC (uS/cm)	580.78 ± 623.08 ^a^	818.53 ± 386.62 ^ab^	921.73 ± 681.36 ^b^	767.17 ± 414.3 ^b^
OC (g/kg)	14.75 ± 10.14 ^a^	6.87 ± 3.66 ^b^	9.73 ± 3.69 ^c^	10.34 ± 3.39 ^c^
OM (g/kg)	25.42 ± 17.49 ^a^	11.84 ± 6.30 ^b^	16.95 ± 6.36 ^c^	17.82 ± 5.84 ^c^
N (g/kg)	3.51 ± 1.38 ^a^	1.53 ± 1.09 ^b^	3.11 ± 1.26 ^a^	2.56 ± 0.65 ^c^
Na (cmolc/kg)	0.063 ± 0.04 ^a^	0.057 ± 0.011 ^bc^	0.059 ± 0.007 ^b^	0.055 ± 0.004 ^c^
K (cmolc/Kg)	0.07 ± 0.02 ^a^	0.07 ± 0.05 ^b^	0.11 ± 0.04 ^a^	0.07 ± 0.02 ^b^
P (g/Kg)	7.38 ± 1.21 ^a^	5.48 ± 1.44 ^b^	17.64 ± 7.25 ^c^	5.81 ± 0.39 ^b^
Ca (cmolc/kg)	0.90 ± 0.51 ^a^	0.42 ± 1.38 ^b^	1.66 ± 0.8 ^c^	0.42 ± 0.15 ^b^
Mg (cmolc/kg)	0.69 ± 0.39 ^a^	0.35 ± 0.22 ^b^	1.02 ± 0.47 ^c^	0.41 ± 0.12 ^b^
Fe (cmolc/kg)	15.89 ± 7.84 ^a^	43.29 ± 17.58 ^b^	32.65 ± 21.38 ^ab^	52.68 ± 26.54 ^b^
Mn (cmolc/Kg)	7.67 ± 3.80 ^a^	2.16 ± 1.41 ^b^	20.54 ± 17.77 ^c^	3.69 ± 2.34 ^b^
Cu (cmolc/kg)	0.23 ± 0.08 ^a^	0.32 ± 0.21 ^b^	0.48 ± 0.33 ^c^	0.25 ± 0.11 ^b^
Zn (cmolc/Kg)	0.65 ± 0.36 ^a^	0.24 ± 0.15 ^b^	1.67 ± 1.74 ^c^	0.37 ± 0.18 ^b^
Al (g/Kg)	5.75 ± 0.39 ^a^	6.88 ± 2.8 ^a^	4.13 ± 3.04 ^b^	8.25 ± 2.12 ^c^
% sand	84.78 ± 3.60 ^a^	78.52 ± 8.95 ^b^	78.82 ± 5.24 ^b^	77.92 ± 7.64 ^b^
% clay	7.20 ± 0.38 ^a^	11.45 ± 1.38 ^b^	10.50 ± 3.23 ^b^	8.68 ± 1.30 ^a^
% silt	8.02 ± 1.38 ^a^	12.39 ± 6.41 ^a^	10.67 ± 3.04 ^ab^	13.4 ± 7.16 ^b^

## Data Availability

The raw data used for this work can be found in the NCBI SRA database in BioProject PRJNA1009150, with the following accession numbers: Agriculture: SRS23561114, SRS23561115, SRS23561126, and SRS23561131 to SRS23561135; Secondary: SRS23561122 to SRS23561125, and SRS23561127 to SRS23561130; Conserved: SRS23561116 to SRS23561121, SRS23561136, and SRS23561137; Preserved: SRS18980979 to SRS18980982.

## Supplementary Materials

The following supporting information can be downloaded at: https://www.mdpi.com/article/10.3390/biology14040390/s1, Table S1: OTU detailed information.
